# A PDE1 inhibitor, vinpocetine, ameliorates epithelial-mesenchymal transition and renal fibrosis in adenine-induced chronic kidney injury in rats by targeting the DNMT1/Klotho/β-catenin/Snail 1 and MMP-7 pathways

**DOI:** 10.1007/s00210-024-03393-0

**Published:** 2024-09-14

**Authors:** Amira Mohammed Abdelfattah, Zeinab A. Mohammed, Aliaa Talaat, Walaa Samy, Mamdouh Eldesoqui, Reham I. Elgarhi

**Affiliations:** 1 Clinical Pharmacology Department, Faculty of Medicine, Zagazig University, Zagazig, Egypt; 2 Forensic Medicine and Clinical Toxicology Department, Faculty of Medicine, Zagazig University, Zagazig, Egypt; 3 Medical Biochemistry Department, Faculty of Medicine, Zagazig University, Zagazig, Egypt; 4 Department of Basic Medical Sciences, College of Medicine, AlMaarefa University, P.O. Box 71666, 11597 Riyadh, Saudi Arabia; 5 Department of Anatomy and Embryology, Faculty of Medicine, Mansoura University, Mansoura, 35516 Egypt

**Keywords:** Vinpocetine, Chronic kidney disease (CKD), Adenine, Epithelial-mesenchymal transition, Klotho, β-Catenin

## Abstract

Tubulointerstitial fibrosis (TIF) is present with chronic kidney disease (CKD). Vinpocetine (Vinpo) is used for treating cerebrovascular deficits, exhibiting some kidney-beneficial effects; however, its role in TIF is uncertain. So, the aim of this study was to investigate its potential impact on adenine-induced fibrotic CKD and explore the underlying mechanistic aspects. Eighteen male Wistar rats were categorized into three groups (*n* = 6 each). Group I was kept as controls and given saline; group II received adenine (300 mg/kg, twice weekly, i.p.) for induction of the CKD model; and group III was administered Vinpo (20 mg/kg/d, orally) concurrently with adenine. All treatments were administered for 4 weeks. Vinpo revealed an improvement in renal function and an alleviation of inflammation triggered by adenine via diminishing serum tumor necrosis factor-α (TNF-α) and interleukin 6 (IL-6) levels. Further, Vinpo repressed the epithelial-mesenchymal transition (EMT) with preserved E-cadherin mRNA expression and lowered gene and immune expression of fibronectin and vimentin, respectively, besides attenuating the elevated G2/M arrest-related molecules (renal Ki67 protein contents and p21 gene expression). Renal pathological alterations caused by adenine were attenuated upon Vinpo administration. Interestingly, Vinpo suppressed abnormal renal β-catenin immunoreactivity, Snail 1, and MMP-7 gene expression while simultaneously restored Klotho protein expression by downregulating DNA methyltransferase 1 enzyme (DNMT1) protein expression in the kidney. These data indicated that Vinpo effectively mitigated EMT and G2/M arrest-induced renal fibrosis in adenine-induced CKD rats by targeting DNMT1-associated Klotho suppression, subsequently inhibiting β-catenin and its fibrotic downstream genes.

**Supplementary Information:**

The online version contains supplementary material available at 10.1007/s00210-024-03393-0.

## Introduction

Tubulointerstitial fibrosis (TIF) is an unavoidable endpoint across all progressive chronic kidney disease (CKD), hastening the disease course towards renal failure (Yuan et al. 2022). TIF is a complicated process characterized by loss of renal parenchymal cells and their replacement by excessive extracellular matrix (ECM) deposits (Nogueira et al. 2017). The contribution of injured renal tubular epithelial cells (TECs) to TIF onset through shedding their epithelial and gaining new distinctive mesenchymal traits in a phenomenon known as epithelial-mesenchymal transition (EMT) has been documented (Hadpech and Thongboonkerd 2024).

The canonical Wnt/B-catenin signaling is integral for the development and sustainability of renal homeostasis (Meng et al. 2020). Despite this pathway being quiet in the normal kidney, it becomes hyperactive upon renal injury, where renal TECs are the basic origin of activated Wnt proteins (Schunk et al. 2021). Upon engagement of Wnt ligands to their receptors, it dephosphorylates B-catenin, triggering its relocation to the nucleus and initiating the transcription of downstream genes (Liu et al. 2022a). This pathway has drawn a lot of attention to date owing to its participation in EMT-driven fibrosis in numerous organs, including renal fibrosis (Li et al. 2021a).

Klotho is a renal tubular epithelium-abundant protein (Prud'homme et al. 2022). It is known to be suppressed in renal diseases, including TIF and renal fibrosis (Li et al. 2018), while increased expression or supplementation of Klotho has been shown to attenuate kidney fibrosis models (Liu et al. 2018; Miao et al. 2021). Klotho functions as an upstream regulator of pathways connected to fibrosis (Tang et al. 2023). Interestingly, Klotho promoter, with CpG abundance sites, is liable to methylation by DNA methyltransferase enzymes (DNMTs), which is one of the molecular bases in epigenetics (Li et al. 2023a), a reversible process that represses gene expression without altering its nucleotide sequence (Berger et al. 2009). In mammals, DNMT-1 is the predominant enzyme for methyltransferase maintenance (Dhe-Paganon et al. 2011) and one of the signal transducers and activators of the transcription 3 (STAT3) pathway’s downstream (Wang et al. 2017). In supporting the importance of Klotho’s epigenetic modulation by methylation in fibrogenesis, Liu et al. (2022c) observed a reduction of aberrant DNMT1 with subsequent restoration of Klotho and alleviation of renal fibrosis in an obstructive nephropathy model.

Vinpocetine (Vinpo), a phosphodiesterase type-1 (PDE1) inhibitor, is clinically recommended for managing diverse cerebrovascular impairments without noteworthy adverse effects noted during its application (Patyar et al. 2011). It has a comprehensive spectrum of properties, like anti-inflammatory, anti-fibrotic (Zhang et al. 2018b), and anti-STAT3 (Kim et al. 2019). Previous research has disclosed Vinpo’s capacity to improve renal function in acute renal damage models (Song et al. 2020; Azouz et al. 2022). Furthermore, Wadie and El-Tanbouly (2017) demonstrated that Vinpo attenuates podocyte damage in diabetic nephropathy. Although Vinpo has some beneficial effects on the kidneys, its significance in relation to TIF is still unclear. So, the current work was undertaken to explore the potential benefits of Vinpo against renal fibrosis in a rat adenine-induced CKD model and clarify the underlying mechanisms.

## Material and methods

### Experimental animals

Eighteen male Wistar rats (weighing between 120 and 200 g) were acquired from the Faculty of Veterinary, Zagazig University, Egypt, and placed in cages in the animal house, Faculty of Medicine, Zagazig University, at an appropriate temperature (25 ± 2 °C), lighting (12-h light/dark cycles), ventilation, and receiving standard nutrition and water. Prior to the study’s beginning, rats were left for a week to become accustomed to the experimental environment. The Animal Ethics Committee of the Faculty of Medicine at Zagazig University approved the experimental protocols and animal handling and care (code approval number: ZU-IACUC/3/F/402/2022).

### Experimental study protocol

The rats were randomly divided into three groups, each containing six rats: the normal control (NC) group, rats were given normal saline (1 mL/rat) via oral gavage; the adenine group, rats were administered an intraperitoneal (i.p.) injection of 300 mg/kg b.w. adenine (Sigma Chemicals, St. Louis, MO, USA) twice weekly to induce CKD (Said et al. 2019); and the adenine + Vinpo group, rats were treated with 20 mg/kg b.w. Vinpo (Sigma Chemicals, St. Louis, MO, USA) daily via oral gavage (Wadie and El-Tanbouly 2017; Elnfarawy et al. 2021) in conjunction with the previously mentioned adenine regimen. All treatments were administered for 4 weeks.

### Sample collection

On the 29th day, blood was drawn from overnight fasting rats’ retro-orbital plexus under the influence of thiopental sodium light anesthesia (5 mg/kg, i.p.) (Onk et al. 2016). The separated serum from the centrifuged blood samples was conserved at − 20 °C until further analysis. Animals were ultimately euthanized through decapitation, with both kidneys removed and split into two parts. The first one was soaked in 10% formalin and prepared for histopathological and immunohistochemistry (IHC) assessment. The second part was homogenized using 0.05 M cold phosphate buffer (pH = 7.4). The generated supernatant following centrifuging homogenates was preserved at − 80 °C pending biochemical and molecular analyses.

### Biochemical assay

#### In serum

Serum urea (Biodiagnostic Kits Cat. No. UR 21 10) and creatinine (Biodiagnostic Kits Cat. No. CR 12 50) were measured spectrophotometrically using the principles of Fawcett and Scott (1960) and Schirmeister et al. (1964), respectively. The TNF-α and IL-6 levels were assessed using the enzyme-linked immunosorbent assay (ELISA) kits (MyBioSource Cat. No. MBS2507393 and MBS264702, respectively), following the kit’s instructions.

#### In renal tissue

Ki-67 was estimated in renal homogenate using the specific ELISA kit (MyBioSource Cat. No.MBS705024). All procedure steps were carried out in compliance with the manufacturers’ guidance.

#### Real-time quantitative polymerase chain reaction (RT-qPCR)

For the isolation of total RNA from homogenized renal tissues in all groups, TRIzol reagent (Invitrogen, Carlsbad, CA, USA) was used. RNeasy mini kits (Qiagen, Valencia, CA, USA) and cDNA kits (iNtRON Biotechnology) were used for the purification of the extracted RNA and the synthesis of cDNA, respectively. The transcript levels of KIM-1, E-cadherin (E-cad), fibronectin (Fn-1), P21, Snail-1, and MMP-7 (the forward [F] and reverse [R] primer sequences are in Table 1) were evaluated via the real-time qPCR technique using an Applied Biosystems instrument on a final volume of 20 µL reaction mixture as follows: 5 min at 95 °C and then 40 cycles (10 s at 95 °C, 60 s at 55 °C, and 10 min at 72 °C). The relative mRNA expression levels of target genes were detected and normalized by endogenous control GAPDH through using the (2^−∆∆CT^) method (Livak and Schmittgen 2001).

**Table 1:** Primer sequences of target genes

Gene	Primers	Accession number
KIM-1	F 5′CGGTGCCTGTGAGTAAATAGAT3′ R 3′CTGGCCATGACACAAATAAGAC5′	AF035963.1
E-cad	F 5′-GGTTTTCTACAGCATCACCG-3′ R 5′-GCTTCCCCATTTGATGACAC-3′	NM_031334.1
Fn-1	F 5′-ATCACCTGGACCCCCGCTCC-3′ R 5′-CGGTTCCCTGCTGCCCGTTT-3′	XM_032901302.1
P21	F 5′-TCTTGCACTCTGGTGTCTCA-3′ R 5′-GGGCTTTCTCTTGCAGAAG-3′	NM_080782.4
Snail-1	F 5′-ATTCTAGACGAGGCTCCCTCTTCCTCTCCA-3′ R 5′-GCTCTAGAAATATCAATAAACTGTACATAT-3′	XM_032902473.1
MMP-7	F 5′-ACTCATGAACTTGGCCACTCTC-3′ R 5′-TTTCCATATAACTTCTGGATGCCT-3′	NM_012864
GAPDH	F 5′-CCTCGTCTCATAGACAAGATGGT-3′ R 5′-GGGTAGAGTCATACTGGAACATG-3′	XM_039082880.1

#### Western blot analysis

Equal amounts of protein samples extracted from renal tissue lysates were loaded onto a 10% SDS–polyacrylamide gel electrophoresis before being transferred to a polyvinylidene fluoride membrane. Following that, the membrane was blocked in Tris-buffered saline with Tween 20 buffer and 3% bovine serum albumin for 1 h and incubated overnight with a primary antibody solution for Klotho (SC-515942, Santa Cruz Biotechnology) and DNMT1 (SC-271729), and then infused with the HRP-conjugated secondary antibody. The chemiluminescent substrate (Clarity TM Western ECL substrate, Bio-Rad cat#170–5060) was applied, and an autoradiograph was imaged. The band intensity of the target proteins against housekeeping beta-actin protein was estimated through image software.

#### Histopathological technique

Following their conservation in 10% neutral-buffering formalin, the collected kidney specimens from each group were dehydrated, cleaned, and embedded in melted paraffin. Using a microtome (Leica®), paraffin Sects. (5 µm) were created and stained with hematoxylin and eosin (H&E) stain and Masson trichrome (MT) stain (Suvarna et al. 2018).

#### Immunohistochemical study

According to Hsu et al. (1981), all experimental groups’ renal sections were stained by IHC using an anti-vimentin antibody [VI-10] (ab20346) and β-catenin (anti-beta catenin antibody (ab6302), Abcam, Cambridge, UK). Briefly, the renal paraffin sections underwent dewaxing, hydration, and staining with the DAB chromogenic agent (Expose mouse and rabbit-specific HRP/DAB detection kit, Abcam; ready-to-use; Cat. #: ab80436). Mayer’s hematoxylin counterstaining was used. All IHC-stained tissue slices were captured using a Swift microscope associated with a Swift digital camera. For quantitative analysis of staining, we selected at least 5 representative areas in total, with both positive cell areas and areas without expression. If a tissue section had areas with both a low and a high abundance of stained cells, both areas were chosen as representative areas and included in the analysis. Individual cells were identified by a strong brown stain and manually counted. The cell counting was repeated three times for each area. All images were analyzed in a blinding fashion.

### Statistical analysis

All data were statistically analyzed and expressed as means ± SD using GraphPad Prism software, version 6 (GraphPad Software Inc., San Diego, CA, USA). The statistical significance differences among experimental groups were carried out by a one-way analysis of variance (one-way ANOVA), followed by a “post hoc” Tukey test. Study correlations were detected using Pearson’s correlation coefficient (r). A *P*-value less than 0.05 was considered significant. In addition, a *P*-value less than 0.001 was defined as highly statistically significant.

## Results

### Effects of Vinpocetine on renal function

**Fig. 1:**
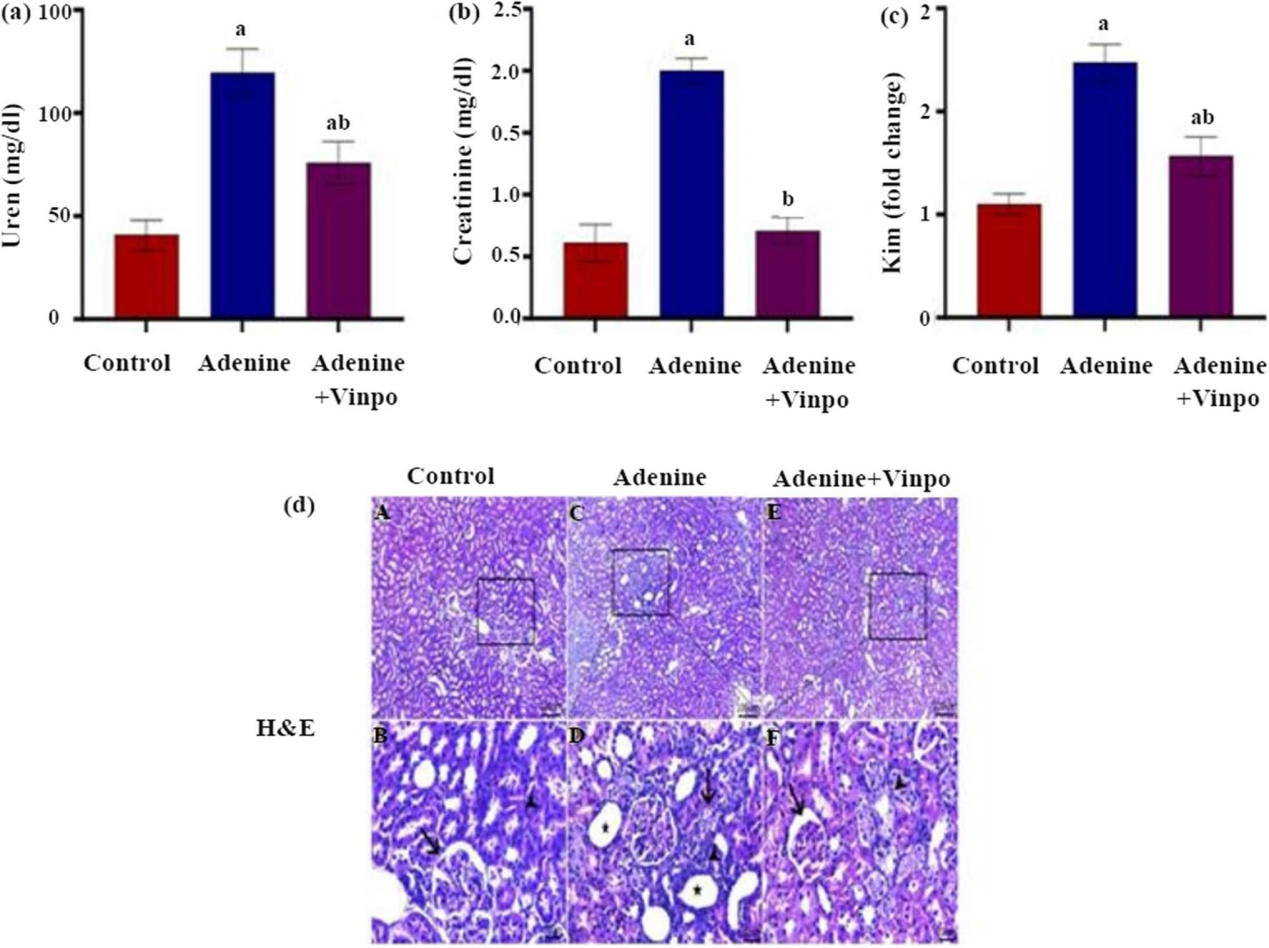
Effects of oral administration of Vinpocetine on renal function and histopathology in adenine-induced CKD in rats. **a** serum urea. **b** serum creatinine. **c** KIM-onefold change. **d** photomicrograph of H&E-stained renal sections (scale bar 20 µm) showing normal histological structures of the glomerular corpuscle (arrow) and renal tubule (arrowhead) in the control group. The focal area of tubulointerstitial fibrosis (arrowhead) is accompanied by a dilated lumen of adjacent renal tubules (star) and some necrotic renal tubular epithelium (arrow) in the adenine group. There is modest hydropic degenerated renal tubular epithelium (arrowhead) and mild shrinkage of glomerular tufts (arrow) in the Vinpo group. Data are expressed as the mean ± SD of different groups (*n* = 6/group). A one-way ANOVA followed by a “post hoc” Tukey test was used for analysis, where ^a^ *P* < 0.001 versus the NC group and ^b^ *P* < 0.001 versus the adenine group

Adenine injection for 4 weeks significantly raised serum urea, creatinine, and KIM-1 mRNA levels compared to the NC group (*P* < 0.001). Meanwhile, rats co-treated with Vinpo exhibited a significant decline in those parameters compared to the rats in the adenine group (*P* < 0.001) (Fig. 1a–c).

### Effects of Vinpocetine on renal histopathological changes

Microscopic examination of H&E-stained renal sections from the NC rats showed typical histological architectures of capillary vessels, glomerular corpuscles, and renal tubules. Conversely, the adenine rats’ renal sections displayed obvious pathological lesions in the form of glomerular atrophy, focal areas of tubulointerstitial fibrosis, and dilated renal tubule lumens. Scattered areas of renal tubular epithelium degeneration and necrosis were also noticed**.** Co-administration of Vinpo with adenine induced ameliorations in the renal histological alterations enhanced by adenine, as illustrated by mildly shrinking glomerular tufts and the presence of a few hydropic degenerated renal tubular epitheliums (Fig. 1d).

### Effects of Vinpocetine on pro-inflammatory cytokines

**Fig. 2:**
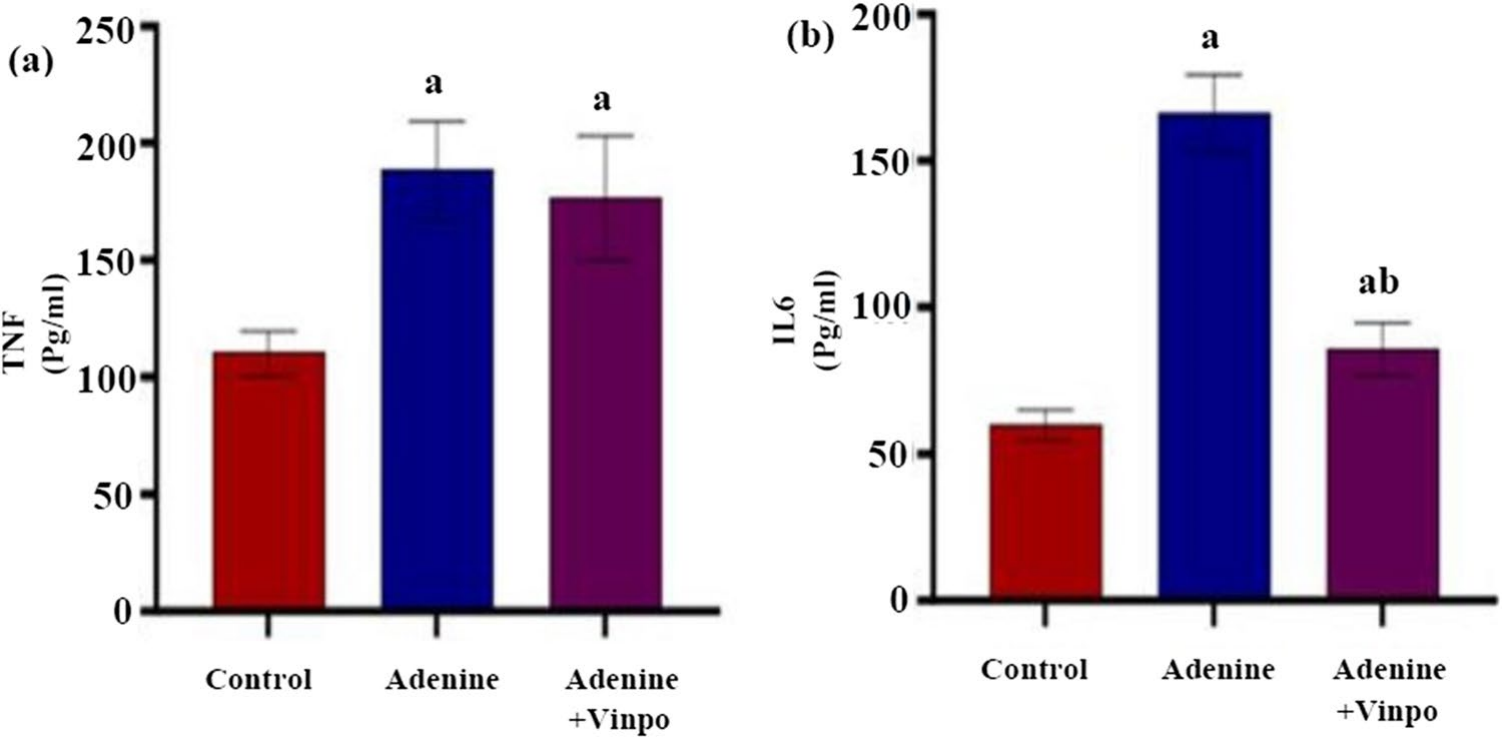
Effect of oral administration of Vinpocetine on serum pro-inflammatory markers in adenine-induced CKD in rats. **a** tumor necrosis factor-α (TNF-α) and (**b)** interleukin-6 (IL-6). Data are expressed as the mean ± SD of different groups (*n* = 6/group). A one-way ANOVA followed by a “post hoc” Tukey test was used for analysis, where ^a^ *P* < 0.001 versus the NC group and ^b^ *P* < 0.001 versus the adenine group

The serum levels of the pro-inflammatory cytokines TNF-α and IL-6 were significantly higher in the adenine-induced CKD rats compared to the normal control rats (*P* < 0.001). The administration of Vinpo with adenine resulted in a significant reduction of IL-6 as compared to the group that received only adenine (*P* < 0.001). Nevertheless, the TNF-α levels remained statistically equivalent to those of the adenine group (*P* > 0.05) (Fig. 2a, b).

### Effects of Vinpocetine on renal fibrosis

**Fig. 3:**
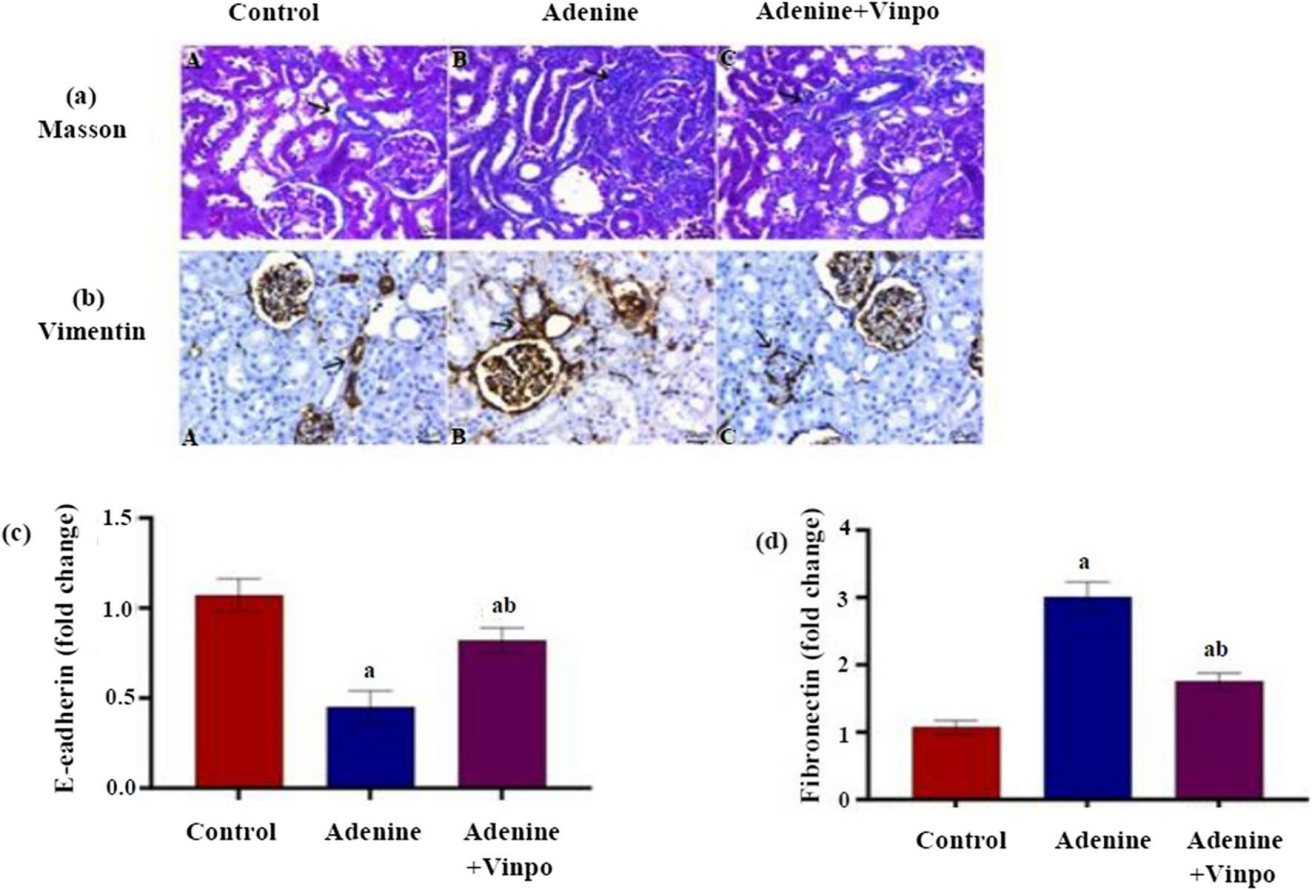
Effects of oral administration of Vinpocetine on renal fibrosis and epithelial-to-mesenchymal transition in adenine-induced CKD in rats. **a** Representative photomicrographs of renal sections stained by Masson’s trichrome (scale bar 20 µm). **b** Representative photomicrographs of vimentin immunohistochemical staining (scale bar 20 µm). Arrows refer to positive-stained cells (the positive-expressed cells revealed a golden-brown colour). **c** E-cadherin fold change and **d** fibronectin fold change. Data are expressed as the mean ± SD of different groups (*n* = 6/group). A one-way ANOVA followed by a “post hoc” Tukey test was used for analysis, where ^a^ *P* < 0.001 versus the NC group and ^b^ *P* < 0.001 versus the adenine group

As depicted in Fig. 3a, the kidney sections stained with MT revealed that the NC group had minimal greenish-blue color staining for collagen fibers. In contrast, the adenine model sections showed abundant staining for collagen deposits within periglomerular tissue and surrounding renal tubules, which was lessened in Vinpo-treated rats. These observations were verified by the statistical analyses of the percentage positive area of collagen visualized by Masson staining in all experimental groups (*P* < 0.001) (Table 2).

**Table 2:** Statistical analysis of the histomorphometric measurements

Morphometric measurements	Groups		
	Control	Adenine	Adenine + Vinpo
Area % of Masson trichrome	4.3 ± 2.5	20.7 ± 2.5^a^	10.7 ± 2.5^ab^
Area % of vimentin	7.3 ± 2.08	26.3 ± 5.13^a^	20 ± 2^ab^
Area % of β-catenin	4.7 ± 1.53	50 ± 5^a^	17.3 ± 3.06^ab^

Data are expressed as the mean ± SD of different groups (*n* = 6/group). A one-way ANOVA followed by a “post hoc” Tukey test was used for analysis, where ^a^*P* < 0.001 versus the NC group and ^b^*P* < 0.001 versus the adenine group

### Effects of Vinpocetine on EMT

Immunohistochemical staining of renal tissues for the EMT protein vimentin was performed, as shown in Fig. 3b. The NC rat’s immunostained renal slice showed weak positive reactions for vimentin only within the vascular walls and glomerular tufts. Strongly positive immunolabeled cells for vimentin in the renal tissue were seen in the adenine group. Contrarily, the adenine + Vinpo group exhibited mild vimentin labeling for a few interstitial cells. The results proven statistically in the adenine-injected rats illuminated a significantly higher percentage of vimentin-positive areas compared to the NC (*P* < 0.001), whereas these results were significantly lower in rats administrating adenine and Vinpo together in contrast to the adenine group (*P* < 0.001) (Table 2).

A significant downregulation in E-cad renal transcript level and an upregulation in renal fibronectin gene expression were recorded in adenine-injected rats compared to the NC (*P* < 0.001). Administration of Vinpo for 4 weeks significantly reversed the changes in renal E-cad as well as fibronectin gene expressions in contrast to the adenine group (*P* < 0.001) (Fig. 3c, d).

### Effects of Vinpocetine on cell cycle arrest

**Fig. 4:**
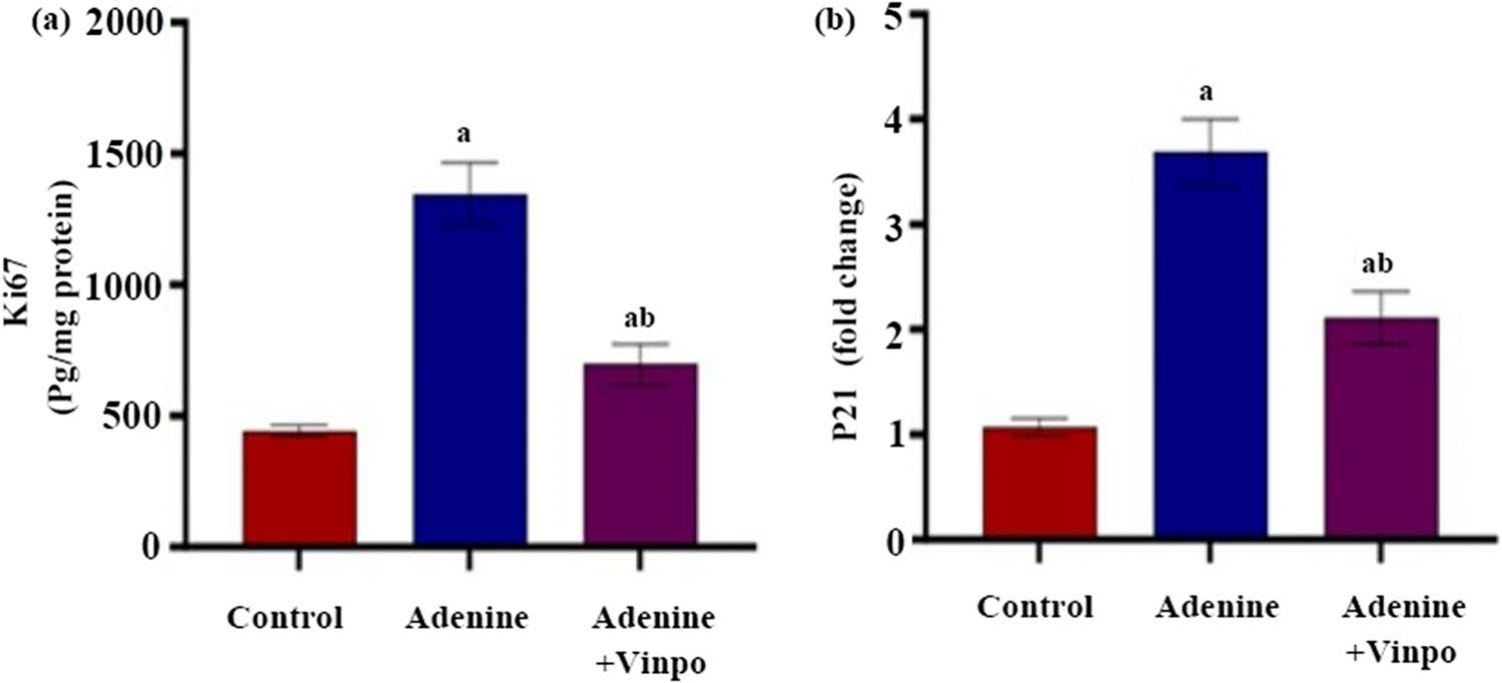
Effects of oral administration of Vinpocetine on renal cell cycle arrest at G2/M stage in adenine-induced CKD in rats. **a** Ki67 protein content. **b** p21 fold change. Data are expressed as the mean ± SD of different groups (*n* = 6/group). A one-way ANOVA followed by a “post hoc” Tukey test was used for analysis, where ^a^ *P* < 0.001 versus the NC group and ^b^ *P* < 0.001 versus the adenine group

The renal content of Ki67 and mRNA expression of P21 in renal tissue were elevated significantly in adenine-injected rats as compared to the NC rats (*P* < 0.001). Four weeks of Vinpo treatment were significantly effective in attenuating the elevated levels of these parameters compared with the adenine group (*P* < 0.001) (Fig. 4a, b).

### Effects of Vinpocetine on β-catenin, Snail-1, and MMP-7

**Fig. 5:**
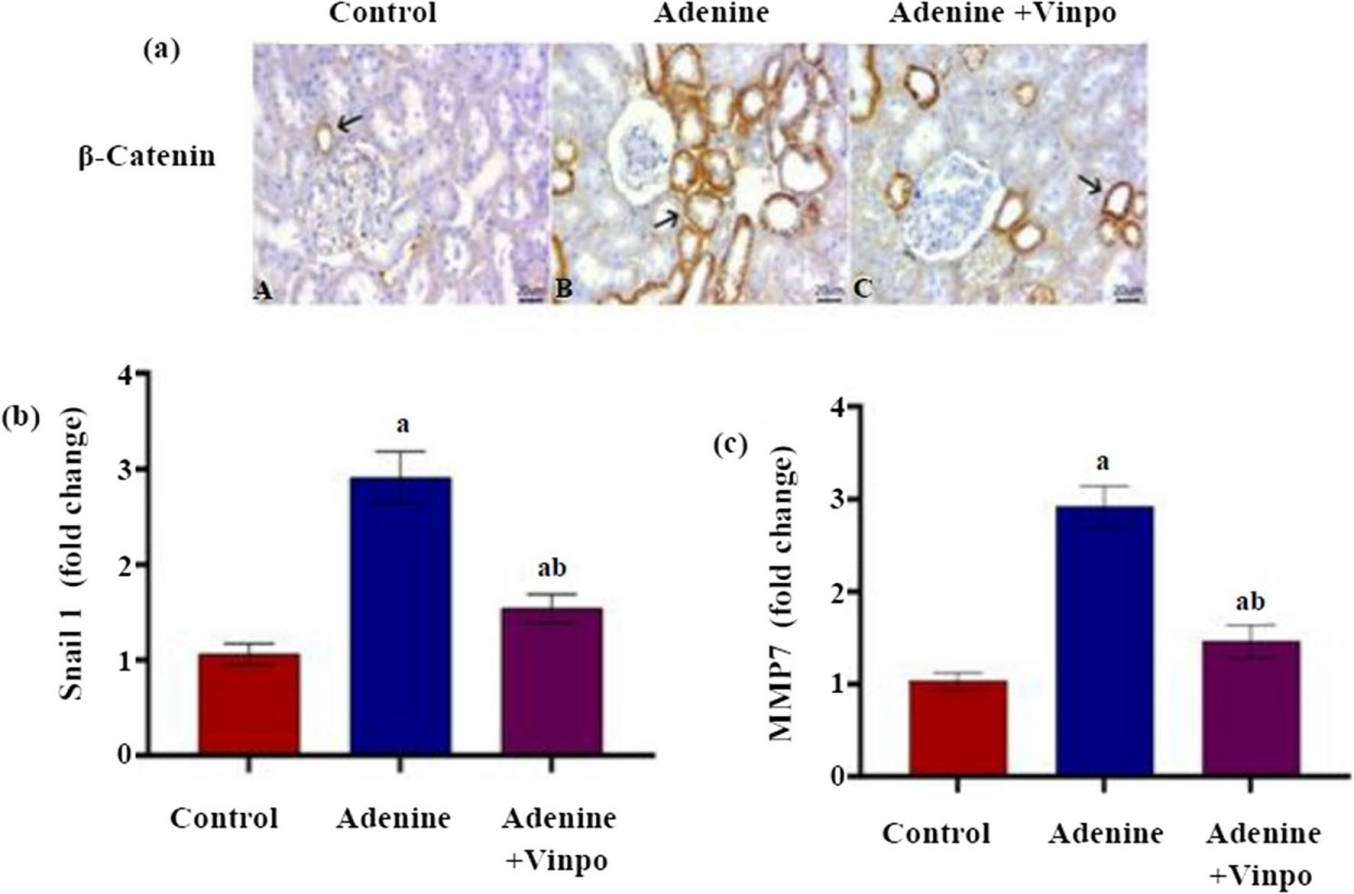
Effects of oral administration of Vinpocetine on β-catenin pathway in adenine-induced CKD in rats. **a** Representative photomicrographs of β-catenin immunohistochemical staining (scale bar 20 µm). Arrows refer to positive-stained cells (the positive-expressed cells revealed a golden brown colour). **b** Snail1 fold change and **c** MMM7 fold change. Data are expressed as the mean ± SD of different groups (*n* = 6/group). A one-way ANOVA followed by a “post hoc” Tukey test was used for analysis, where ^a^ *P* < 0.001 versus the NC group and ^b^ *P* < 0.001 versus the adenine group

Following adenine administration, the β-catenin pathway was reactivated, as affirmed by strong membranous and cytoplasmic β-catenin staining in a large number of renal tubular epithelium in the adenine rat’s renal section compared to few β-catenin staining of some renal tubular epithelium in the NC section. The labelled cells containing β-catenin golden brown staining were attenuated in the adenine + Vinpo group. Statistical evaluations of the percentage of β-catenin-positive staining areas among different groups confirmed these findings (*P* < 0.001) (Fig. 5a, Table 2).

Renal tissue extracted from adenine-induced CKD rats had a significant upregulation of Snail-1 and MMP-7 transcript levels compared to the NC group (*P* < 0.001). On the contrary, co-treatment with Vinpo at a dose of 20 mg/kg for 4 weeks significantly diminished the alternations caused by adenine in Snail-1 and MMP-7 (*P* < 0.001) (Fig. 5b, c).

### Effects of Vinpocetine on renal Klotho

**Fig. 6:**
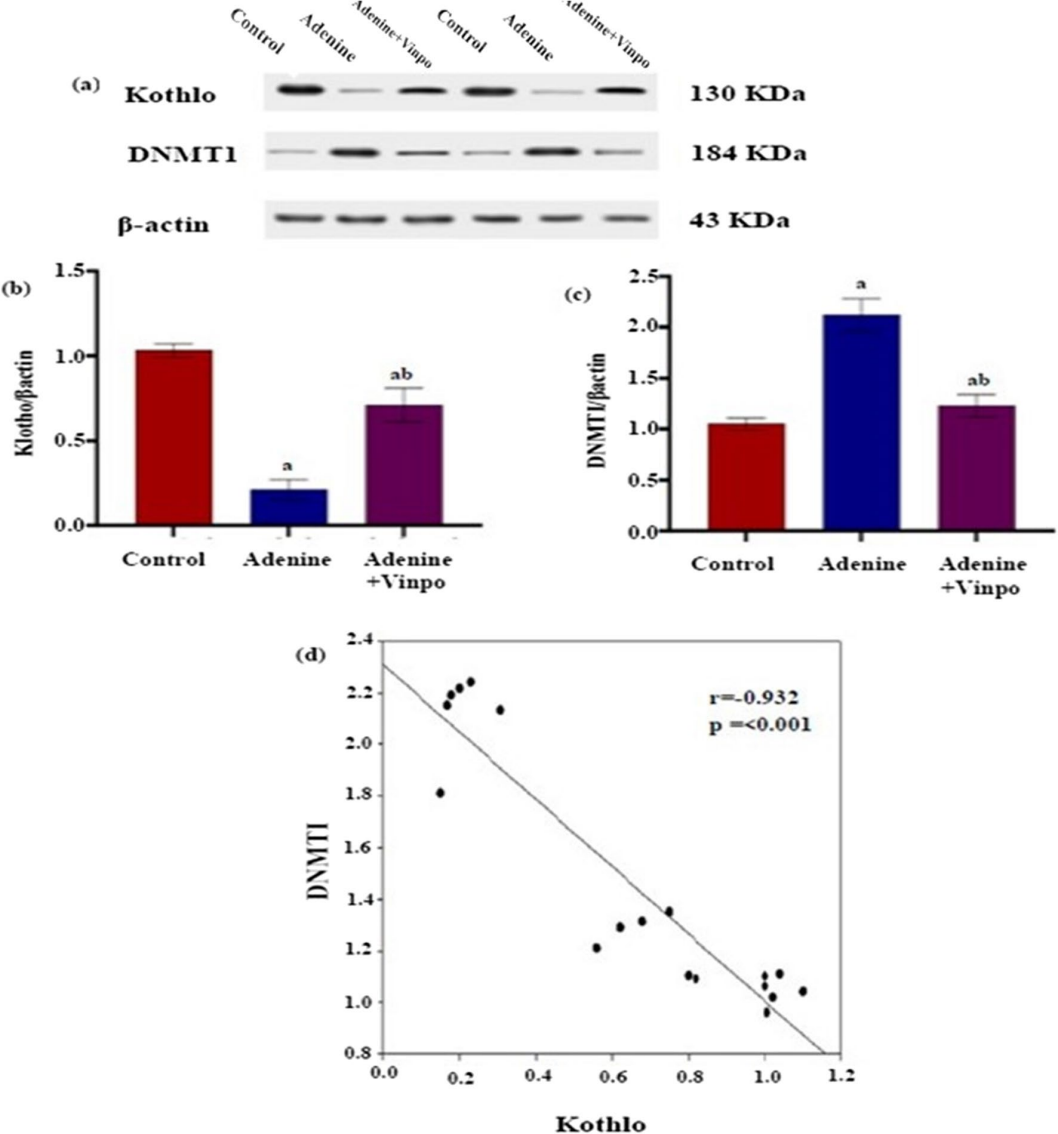
Effect of oral administration of Vinpocetine on renal Klotho and DNMT1 in adenine-induced CKD in rats. **a** Klotho and DNMT1 proteins were evaluated by western blotting. **b** Quantitative analysis of western blotting of Klotho. **c** Quantitative analysis of western blotting of DNMT1. Data are expressed as the mean ± SD of different groups (*n* = 6/group). A one-way ANOVA followed by a “post hoc” Tukey test was used for analysis, where ^a^ *P* < 0.001 versus the NC group and ^b^ *P* < 0.001 versus the adenine group. **d** Correlation between renal DNMT1 and Klotho

The renal Klotho protein expression in the adenine rats was significantly downregulated compared to that in the NC (*P* < 0.001). On the other hand, treating adenine-injected rats with Vinpo significantly upregulated renal Klotho protein expression compared to the adenine group (*P* < 0.001) (Fig. 6a and b).

### Effects of Vinpocetine on DNMT1

The kidneys of the adenine-treated rats showed a significant upregulation of the protein DNMT1 expression compared to the NC (*P* < 0.001). On the other hand, daily oral administration of Vinpo for 4 weeks significantly decreased the amount of DNMT1 protein in the kidneys compared to the adenine group (*P* < 0.001) (Fig. 6a and c). Renal DNMT1 protein expression showed a strong negative correlation with renal klotho protein expression (*r* =  − 0.932, *p* < 0.001) (Fig. 6d).

## Discussion

The current work presents Vinpo therapy’s beneficial effects on TIF in an adenine-induced fibrotic CKD rat model, presumed via its ability to inhibit DNMT1-mediated Klotho suppression in the kidney. Furthermore, β-catenin and its downstream signaling genes were suppressed, coupled with EMT and G2/M cell cycle arrest markers, owing to the heightened Klotho protein expression in the presence of Vinpo. Our outcomes imply that Vinpo alleviated EMT-induced TIF by targeting DNMT1/Klotho/β-catenin/Snail1 and the MMP-7 pathway.

Adenine (300 mg/kg, i.p.) was used in the present work to induce CKD-related renal fibrosis. Unlike other experimental models, it is simple and easy to use. It more precisely captures the pathophysiological alterations in clinical CKD, making it suited for testing prospective anti-fibrotic drugs (Said et al. 2019).

In this study, worsening conventional renal functional tests (higher serum urea and creatinine levels) served as evidence of adenine-induced renal injury. Moreover, adenine-induced renal damage was linked to a dramatic rise in the gene expression of KIM-1, which is more specific than conventional markers in reflecting renal tubular damage **(**Vaidya et al. 2010). Additionally, histological analysis of adenine rats’ renal tissues revealed tubular lumen dilatation with areas of tubulointerstitial fibrosis, shedding renal tubular epithelium, and excessive glomerular atrophy, confirming injuries brought on by adenine. These results are in line with prior studies (Gori et al. 2021; Hayeeawaema et al. 2023) and may be attributable to the deposition of excess adenine and its metabolites in renal tubules, where they stimulate crystal formation, resulting in tubular blockage, secondary inflammatory load, fibrosis, and eventually renal failure (Diwan et al. 2018). In this experiment, oral administration of Vinpo caused an improvement in renal function parameters as well as renal pathological alterations triggered by adenine, supporting its nephroprotective properties published earlier (Abbas et al. 2020b; Azouz et al. 2022)*.*

Inflammation has been recognized as one of the prevalent traits of CKD (Kadatane et al. 2023). The interleukin family and TNF-α collaborate to create a long-lasting network of inflammation that is undoubtedly essential for the pathogenesis of this disorder (Sarkaki et al. 2023; Amini et al. 2024). In our research, TNF-α and IL-6 levels were increased in the adenine rats’ serum, as reported earlier (Xiao et al. 2021; Ashour et al. 2023). Treatment with Vinpo in this work displayed an anti-inflammatory impact, as demonstrated by attenuating the elevated serum pro-inflammatory cytokines (TNF-α and IL-6) brought on by adenine. Our outcomes align with those of Fattori et al. (2017) and Song et al. (2020).

Renal TECs are the primary constituents of the renal parenchyma and are often the most important target of kidney damage (Zhou and Liu 2016). In fibrogenesis, injured renal TECs undergo EMT, a process where damaged renal TECs transform into mesenchymal ones with the production of more myofibroblast-like cells (Hadpech and Thongboonkerd 2023). It has been reported that around one-third of activated myofibroblasts originate from the EMT of renal tubules (Iwano et al. 2002). Moreover, a positive relationship between EMT levels and renal fibrogenesis degree has been documented (Schelling 2016), whereas the extent of fibrotic lesions improved upon suppressing the EMT in different kidney models (Wei et al. 2022; Wu et al. 2023). The primary stage in this phenomenon is the loss of E-cad, which is a key epithelial element that is necessary for intercellular adhesion junctions, mobility, and proliferation of epithelial cells (Zhou et al. 2022a). Another valuable indicator in this process is vimentin, an intermediate filament protein located in mesenchymal cells (Wang et al. 2018). Fibronectin is also typically used as an EMT biomarker due to its lack of normal epithelium (Hu et al. 2022). In harmony with the results of Lin et al. (2023), we uncovered additional evidence that EMT and TIF were provoked in the adenine kidney model. Notably, the favorable effect of Vinpo on renal fibrosis and EMT was affirmed in our research. Vinpo administration for 4 weeks diminished collagen fiber deposition, upregulating epithelial markers (E-cad), together with a decline in mesenchymal markers (vimentin and fibronectin). These findings imply that Vinpo could reduce TIF by inhibiting the EMT in the renal tubular epithelium.

There is mounting evidence highlighting the implications of renal TECs’ cell cycle arrest in CKD fibrogenesis. Following a kidney injury, the cell cycle of the renal TECs was halted in the G2/M stage, restricting their regenerative capacity and encouraging collagen deposition by activating myofibroblasts (Zhang et al. 2023). Of particular interest, reversing the arrest of TECs in the G2/M reduced fibrosis, confirming a direct mechanistic connection between fibrosis and renal tubules’ G2/M arrest (Feiteng et al. 2022). It is worth noting that the renal tubules’ G2/M arrest is the biological outcome of renal tubular EMT (Lovisa et al. 2015). Accordingly**,** we investigated the Vinpo effect on EMT-induced cell cycle arrest. P21, a cyclin-dependent kinase inhibitor, is one of the key G2/M cell cycle arrest mediators (Zhao et al. 2020). Ki-67, a proliferative activity marker that is expressed throughout cell cycle phases except G0 (Juríková et al. 2016), is a reliable indicator of proliferating interstitial myofibroblasts (Zhang et al. 2018a). In line with the preceding research by Abbas et al. (2020a) and Li et al. (2023b), we reported G2/M cell cycle arrest in adenine kidney rats. Treating rats with Vinpo for 4 weeks markedly mitigated signs of adenine-induced G2/M arrest (reduced Ki-67 protein level and p21 gene expression), suggesting that Vinpo alleviated renal fibrosis via attenuating EMT and EMT-induced G2/M arrest. In a hepatic fibrosis rat, Elnfarawy et al. (2021) stated Vinpo’s anti-proliferative and anti-fibrotic effects on Ki-67.

In order to figure out the mechanistic aspect of Vinpo in controlling EMT-driven TIF, the role of β-catenin signaling was studied. There is growing data emphasizing that canonical β-catenin is one of the fundamental EMT process inducers (Liu et al. 2022b). Following Wnt activation, degradation of the cytoplasmic β-catenin complex causes loss of the β-catenin/E-cad interaction complex, subsequently beginning the EMT procedure (Tian et al. 2011). Likewise, the pathway’s master effector, active β-catenin, is relocated into the nucleus, which induces batteries of EMT-related downstream genes (Hu et al. 2022). Snail1 is one of the essential downstream transcription genes of β-catenin that facilitates the initial stage of EMT by repressing E-cad transcription (Xie et al. 2020). Also, MMP-7 is another β-catenin transcription gene that encourages EMT by causing E-cad to degrade (Zhou et al. 2017). La et al. (2018) recorded an increase in canonical β-catenin in the adenine-induced CKD rats. Similarly, in this research, adenine injection enhanced the renal β-catenin immunoreactivity, which, in turn, drove the expression of its downstream genes encoding Snail1 as well as MMP-7. Conversely, Vinpo-treated rats effectively remitted these alterations, providing that Vinpo elicited suppression of EMT-associated G2/M arrest and renal fibrosis, likely through its inhibitory effect on the β-catenin/Snail1 and MMP7 pathways.

The membrane-bound renal protector protein Klotho is plentiful in the kidney and has a plethora of functions, including anti-fibrosis (Olejnik et al. 2018). Klotho deficit appears to be a promising CKD-sensitive biomarker because its deficit exacerbates the onset and progression of CKD (Zou et al. 2018). Liu and his colleagues (2017) noticed that Klotho treatment suppressed EMT and correlated renal fibrosis in cyclosporine A rats. Most importantly, Li et al.’s (2021b) research found that Klotho inhibition activated β-catenin with subsequent acceleration of renal EMT and fibrosis in a renal transplant model. Klotho protein directly sequesters active Wnt ligands and prevents their binding to the receptor, thereby interfering with the subsequent downstream pathway (Satoh et al. 2012). Presently, we provided further evidence that Klotho was downregulated in the adenine kidney, in agreement with that of Zhao et al. (2021). Our data pointed out the first evidence of the ability of Vinpo to up-regulate renal Klotho protein expression in the adenine-induced CKD rat model. Collectively, Klotho activation by Vinpo was thought to be responsible for Vinpo’s counteracting effect on β-catenin signaling-induced renal EMT-associated TIF and G2/M arrest and might be speculated as a mechanism for its beneficial influence on renal fibrosis.

One of the vital factors controlling Klotho expression is the DNA hypermethylation of its CpG-rich promoter. Emerging literature suggests the involvement of Klotho promoter hypermethylation in the pathogenesis of Klotho deficit and fibrotic kidney disease (Yang et al. 2020; Zhou et al. 2022b). Subsequently**,** we determined that renal DNMT1’s protein expression was upregulated in the adenine kidney, and this negatively correlated with the suppression of Klotho’s protein expression, similar to the result of Zhang et al. (2016). Nevertheless, this was drastically cured with Vinpo. The decrease in DNMT1 expression contributed to a reduction in CpG promoter gene hypermethylation, which in turn activates the translation of these genes. In light of these, we could deduce that Vinpo restored Klotho expression and offered renal protection by inhibiting DNMT1. As we illuminated, Vinpo restored the Klotho loss via epigenetic modulations of DNMT1; we hypothesized that Vinpo’s demethylating properties might be due to its STAT3 inhibitory effect. Vinpo is a well-known STAT3 inhibitor (Kim et al. 2019; Elwany et al. 2023). It has been established that STAT3 over-activation is linked to fibrotic CKD (Zheng et al. 2020). Intriguingly, one of the STAT3 pathway’s downstream targets is DNMT1 (Wang et al. 2017). Hence, inhibition of STAT3 by Vinpo affects DNMT-1 expression, although this point requires further investigation.

In conclusion, this research shows that Vinpo effectively combats TIFs in adenine-induced CKD in rats by mitigating the EMT process and G2/M cell cycle arrest. This may be due to suppressing DNMT1 expression, which in turn restores Klotho expression in the kidney and subsequently counterbalances the action of β-catenin and its fibrotic downstream targets (Snail 1 and MMP7). We anticipate that our findings could offer valuable guidance for clinical trials.

## Supplementary Information

Below is the link to the electronic supplementary material.

Supplementary file1 (JPG 12 KB)

Supplementary file2 (JPG 11 KB)

Supplementary file3 (JPG 12 KB)

## Data Availability

No datasets were generated or analysed during the current study.
