# Supplementary figures and images for: A PDE1 inhibitor, vinpocetine, ameliorates epithelial-mesenchymal transition and renal fibrosis in adenine-induced chronic kidney injury in rats by targeting the DNMT1/Klotho/β-catenin/Snail 1 and MMP-7 pathways
Source: Naunyn Schmiedebergs Arch Pharmacol. 2024 Sep 14;398(3):2769–81. doi: 10.1007/s00210-024-03393-0 (PMC11919975; doi:10.1007/s00210-024-03393-0)

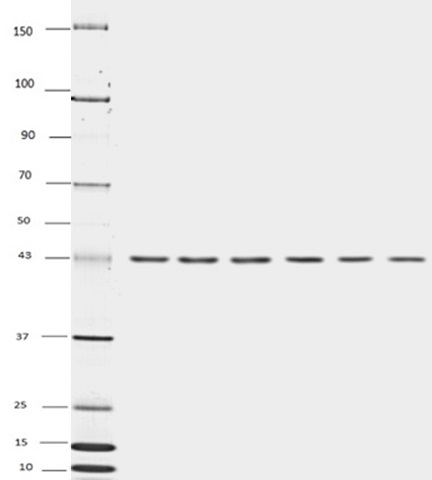

Supplement: Supplementary file 1 — Supplementary file1 (JPG 12 KB) [file 210_2024_3393_MOESM1_ESM.jpg]

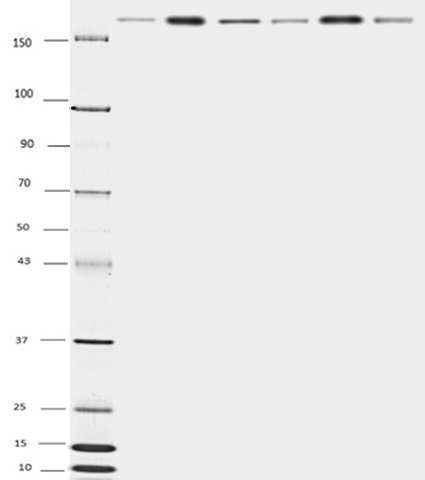

Supplement: Supplementary file 2 — Supplementary file2 (JPG 11 KB) [file 210_2024_3393_MOESM2_ESM.jpg]

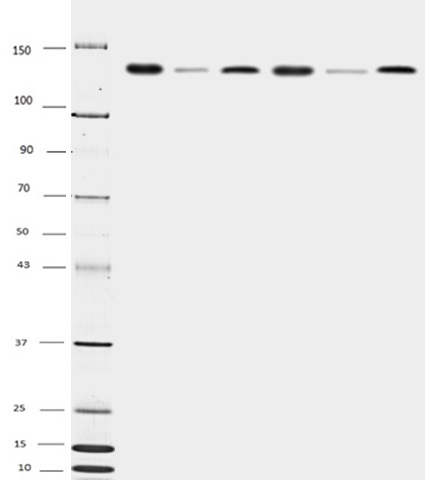

Supplement: Supplementary file 3 — Supplementary file3 (JPG 12 KB) [file 210_2024_3393_MOESM3_ESM.jpg]
